# Validation of a Low-Cost Open-Ended Coaxial Probe Setup for Broadband Permittivity Measurements up to 6 GHz

**DOI:** 10.3390/s25133935

**Published:** 2025-06-24

**Authors:** Julia Arias-Rodríguez, Raúl Moreno-Merín, Andrea Martínez-Lozano, Germán Torregrosa-Penalva, Ernesto Ávila-Navarro

**Affiliations:** Elche Microwave Laboratory (EMwLab), Engineering Research Institute of Elche (I3E), Miguel Hernández University of Elche, 03202 Elche, Spain; raul.morenom@umh.es (R.M.-M.); andrea.martinezl@umh.es (A.M.-L.); gtorregrosa@umh.es (G.T.-P.); eavila@umh.es (E.Á.-N.)

**Keywords:** complex permittivity, open-ended coaxial probe, low-cost system, SMA-based dielectric sensor, microwave measurement, dielectric spectroscopy, calibration, reproducibility

## Abstract

This work presents the validation of a low-cost measurement system based on an open-ended coaxial SMA (SubMiniature version A) probe for the characterization of complex permittivity in the microwave frequency range. The system combines a custom-fabricated probe, a vector network analyzer, and a dedicated software application that implements three analytical models: capacitive, radiation, and virtual transmission line models. A comprehensive experimental campaign was carried out involving pure polar liquids, saline solutions, and biological tissues, with the measurements compared against those obtained using a high-precision commercial probe. The results confirm that the proposed system is capable of delivering accurate and reproducible permittivity values up to at least 6 GHz. Among the implemented models, the radiation model demonstrated the best overall performance, particularly in biological samples. Additionally, reproducibility tests with three independently assembled SMA probes showed normalized deviations below 3%, confirming the robustness of the design. These results demonstrate that the proposed system constitutes a viable alternative for cost-sensitive applications requiring portable or scalable microwave dielectric characterization.

## 1. Introduction

The accurate characterization of the complex permittivity of materials in the microwave frequency range is essential for a wide range of scientific, industrial, and biomedical applications. Dielectric properties are intrinsically linked to the molecular composition, water content, and ionic conductivity of materials, making them relevant for noninvasive diagnostics, industrial quality control, and electromagnetic compatibility testing. Accordingly, there is growing interest in broadband, efficient, and reliable measurement techniques capable of capturing the frequency-dependent dielectric response of a wide variety of samples. Several comprehensive reviews have been published covering the state of the art in microwave permittivity measurement, including both resonant and non-resonant techniques [1,2], with more recent works emphasizing sensor design strategies [3] and emerging applications, such as microfluidics, metamaterials, or artificial intelligence (AI)-assisted data interpretation [4]. These surveys highlight a sustained demand for broadband, low-cost solutions for dielectric characterization—objectives that this work directly addresses.

A variety of measurement techniques have been developed for determining complex permittivity, including resonant cavity methods, transmission/reflection line techniques, free-space setups, and open-ended coaxial probes. Among these, the latter have become especially popular due to their broadband coverage, non-destructive nature, and minimal sample preparation requirements [5,6]. They are particularly well suited for liquids, soft solids, and biological tissues. It relies on measuring the complex reflection coefficient at the tip of a coaxial line in contact with the material under test (MUT) and extracting the permittivity via analytical or numerical models that account for fringing fields, radiation effects, and wave propagation phenomena [7,8,9].

In biomedical contexts, open-ended coaxial probes are widely used for tissue discrimination, cancer diagnostics, hydration or glucose assessment, and treatment monitoring [10,11,12,13,14]. They are employed both in vivo and ex vivo, using biological or artificial tissues, and support clinical applications, such as thermal ablation or dielectric imaging [15,16,17,18]. A similar utility has been demonstrated in food quality control and agriculture, where dielectric spectroscopy enables moisture estimation, ripeness analysis, and adulteration detection in a wide variety of perishables [19,20,21,22,23]. The technique is also relevant in pharmaceutical formulation, process monitoring in polymer manufacturing, and materials research [24,25,26,27].

The foundational work of Stuchly and Stuchly [10] established the open-ended coaxial probe method for broadband dielectric spectroscopy. This was subsequently refined through improved probe modeling [5,6], calibration techniques [9], and numerical tools [18,28]. The calibration of coaxial probes remains a critical step in ensuring measurement accuracy. Standard three-point calibrations—typically using air, a short circuit, and distilled water—are employed to reference the probe response and correct for systematic errors [6,9,18]. These standards provide known admittance or permittivity values that anchor the error model and ensure the traceability of the results. More sophisticated calibration protocols have also been proposed, including time-domain de-embedding, multiple-load reflection methods, and Monte Carlo error estimation [9,18,28]. Probe design and calibration methods play a pivotal role in measurement accuracy. Comprehensive reviews have provided guidelines for effective calibration using Short-Open-Load (SOL) methods and similar techniques [9,29]. Advanced computational tools have further streamlined data analysis and modeling, making these methods more accessible to researchers [28]. For example, the modeling of coaxial probes using capacitive-load and radiation models has been refined over the years to improve measurement precision and reliability [30]. Additionally, innovative approaches, such as time–decay analysis, have been proposed to track and compensate for sample-property drift in lossy phantoms during prolonged measurements [31]. Furthermore, methods for predicting the sensing radius of a coaxial probe, based on its dimensions, have been developed, which are particularly useful for optimizing probe placement in various materials and improving the accuracy of measurements [32].

Despite the advances in calibration protocols and software accessibility, the adoption of dielectric spectroscopy using open-ended coaxial probes is still limited by the high cost of commercial systems. These typically rely on high-end VNAs, precision-machined probes, and proprietary software suites [29,33,34,35], which restrict their use in resource-constrained environments, such as educational institutions, small laboratories, and low-income regions. The cost of commercial dielectric probes alone can exceed several hundred euros, excluding the VNA and software licenses. Furthermore, their mechanical complexity and calibration demands may reduce portability and limit suitability for field applications.

To overcome these barriers, there has been a growing focus on low-cost solutions, including several that repurpose standard SubMiniature version A (SMA) connectors [14,36,37,38]. These studies demonstrate that, with proper calibration and modeling, such probes can offer surprisingly accurate results in applications ranging from food analysis to tissue characterization. Notable contributions include slim coaxial sensors for rice-grain moisture sensing [36], modified SMA connectors for detecting water adulteration in honey and latex [38], and custom probes for liquid food analysis [37]. In the biomedical domain, a recent study [14] used a virtual line model to estimate the dielectric properties of biopsy-excised tissues, supporting the viability of simplified systems in clinical workflows.

In parallel, compact, low-cost vector network analyzers (VNAs)—often USB- or PC-driven—have become more prevalent, enhancing the practicality of do-it-yourself coaxial probe setups [39]. Python-based software (e.g., using PyVISA, NumPy, Matplotlib) can manage data acquisition in real time, providing immediate permittivity results while allowing users to customize or inspect the underlying code.

Yet, the accuracy of these systems critically depends on the electromagnetic model used for S_11_ interpretation, on the quality of the calibration, and on the degree of radiation losses at high frequencies. While simple capacitive models may suffice at low frequencies—especially for low-loss or weakly conductive samples—more advanced models, such as the radiation and virtual line models, offer better performance over broader ranges by incorporating wave propagation and radiative phenomena. Studies by Berube et al. [7] and Gajda and Stuchly [8] have benchmarked multiple models, emphasizing trade-offs between accuracy and computational complexity. The optimal model depends on the sample permittivity, frequency range, and probe geometry.

Many previous works involving custom coaxial probes have targeted specific sample types or frequency bands and have typically relied on a single modeling strategy. Some studies prioritized food or agricultural applications, such as rice-grain moisture estimation using polynomial fits [36] or food liquid characterization with univariate and Partial-Least-Squares (PLS) regression [34]. Others focused on biomedical contexts, applying the virtual line model to biopsy-excised tissues without comparisons to alternative approaches [14]. In several cases, the analysis was constrained to narrow frequency bands, as in studies on honey and latex [38], or tailored to specific mixtures, such as alcoholic beverages characterized up to 20 GHz using a simplified calibration set [40]. The geometry of the probe also influences its performance: slim coaxial lines (e.g., UT-085) can reduce radiative effects [17], whereas wider geometries, like the type-N probe used in [41] for cell membrane analysis, offer a lower bandwidth. One example used an RG405-based probe with the virtual line model, operating up to 3 GHz, but with limited calibration complexity and a narrower frequency range compared to the system presented in this work [39]. Together, these studies highlight the diversity of experimental strategies, but also the lack of comprehensive comparisons across models, geometries, and sample classes within a unified system.

In contrast to these prior efforts, this work offers a multi-pronged contribution. It validates a low-cost dielectric measurement system based on an open-ended SMA probe, a compact VNA, and a custom Python application capable of processing reflection data using three modeling approaches: capacitive, radiation, and virtual transmission line. Unlike previous studies, we assess the performance of each model across increasingly complex sample types—pure polar liquids, saline solutions, and biological tissues—within a unified experimental framework and benchmark all the results against a high-precision commercial system. Moreover, this study stands out by including a reproducibility analysis across three independently built probes, assessing the variability in dielectric extraction due solely to fabrication dispersion—an aspect rarely addressed in the prior literature.

The remainder of the paper is organized as follows: Section 2 reviews the theoretical models and calibration approaches for permittivity extraction. Section 3 outlines the experimental materials, instrumentation, and measurement protocol. Section 4 presents and discusses the results, including a breakdown by material category and reproducibility evaluation. Finally, Section 5 provides concluding remarks and suggestions for future work.

## 2. Theoretical Foundations

### 2.1. Complex Permittivity

When an external electric field is applied to a material, polarization occurs as positive and negative charges separate or shift in response to the field. In conductive materials, charges are free to move, but in dielectrics, they are bound to their atoms or molecules and can only partially reorient. As a consequence, a material subjected to an electric field develops dipoles. The overall response of a dielectric material to an applied alternating electric field is expressed in terms of its complex permittivity, $\varepsilon^{*}$, defined as

(1) $$\varepsilon^{*}=\varepsilon^{\prime }-j\varepsilon^{\prime \prime },$$

where the real part, $\varepsilon^{\prime }$, quantifies the ability of a material to store electric energy (i.e., its dielectric constant), whereas the imaginary part, $\varepsilon^{\prime \prime }$, accounts for the dielectric losses arising from the lag in the polarization response and conduction phenomena.

In dielectrics, several mechanisms contribute to polarization. Electronic polarization arises when the external field displaces electron clouds relative to their nuclei. Due to the very small mass of electrons, this process is nearly instantaneous and becomes dominant at optical and higher frequencies. Atomic polarization occurs due to the displacement of entire atoms or molecular groups. Being slower than electronic polarization (due to the larger mass involved), atomic polarization becomes significant in the infrared and far-infrared ranges. Dipolar (orientational) polarization is characteristic of materials with permanent dipoles. Under an applied field, these dipoles align with the field direction, but thermal motion counteracts that alignment, making this mechanism strongly dependent on both frequency and temperature. Dipolar polarization is particularly important at microwave frequencies in polar liquids such as water, where molecular inertia and viscosity produce a noticeable phase lag. Interfacial polarization (or space charge polarization) arises at boundaries between materials with differing electrical properties. In heterogeneous materials—like biological tissues—charge carriers accumulate at these interfaces (Maxwell–Wagner polarization), creating an additional polarization effect, especially at lower frequencies (typically below 1 MHz).

Furthermore, besides these polarization mechanisms, ionic conduction plays a notable role in the dielectric response. In biological tissues, for instance, mobile ions (e.g., Na^+^, K^+^, Ca^2+^, Cl^−^) contribute to energy losses, thereby increasing the imaginary component of the permittivity.

In materials where dipolar polarization is the predominant mechanism, the frequency dependence of $\varepsilon^{*}$ is often described by the Debye relaxation model. Assuming the relaxation process can be approximated by a single time constant, *τ*, the Debye expression is given by [42]

(2) $$\varepsilon^{*}\left(\omega \right)=\varepsilon_{\infty }+\frac{\varepsilon_{s}-\varepsilon_{\infty }}{1+j\omega \tau },$$

where $\varepsilon_{s}$ is the static permittivity measured at low frequencies (when dipoles have enough time to fully align with the field), and $\varepsilon_{\infty }$ is the permittivity at high frequencies where slower polarization mechanisms, such as dipolar orientation, cannot follow the applied field, and only fast responses like electronic polarization contribute. Here, ω is the angular frequency, and τ is the characteristic relaxation time associated with the dipolar process.

Many real materials, however, do not exhibit a single, uniform relaxation but rather a distribution of relaxation times due to structural inhomogeneities and multiple overlapping mechanisms. In these situations, a more general approach is provided by the extended Cole–Cole equation [43], which explicitly accounts for ionic conduction [44]:

(3) $$\varepsilon^{*}\left(\omega \right)=\varepsilon_{\infty }+\sum_{k}\frac{\varepsilon_{s,k}-\varepsilon_{\infty ,k}}{1+{\left(j\omega \tau_{k}\right)}^{1-\alpha_{k}}}-\frac{j\sigma_{i}}{\omega \varepsilon_{0}}.$$

Here, each term in the summation corresponds to a distinct relaxation, labeled by *k*. For each process, $\varepsilon_{s,k}$ is the static permittivity, $\varepsilon_{\infty ,k}$ is the high-frequency limit, and $\tau_{k}$ is the relaxation time. The parameter $\alpha_{k}$ (with $0\leq \alpha_{k}<1$) captures the broadening of the relaxation time distribution ($\alpha_{k}=0$ recovers the Debye behavior). The final term, −$j\sigma_{i}$/$\left(\omega \varepsilon_{0}\right)$, includes the contribution of ionic conductivity, ($\sigma_{i}$), with $\varepsilon_{0}$ being vacuum permittivity.

Thus, while the Debye model provides a straightforward description for systems dominated by a single dipolar relaxation, the extended Cole–Cole formulation is needed for materials exhibiting multiple overlapping relaxation processes and significant conductive losses. By fitting experimental data to these models, one can extract parameters that quantitatively describe the dielectric response over a broad frequency range.

### 2.2. Open-Ended Coaxial Probe Method

The open-ended coaxial probe technique is a well-established, broadband, noninvasive method for determining a material’s complex permittivity. It allows for the precise electromagnetic characterization of a wide range of media, including biological tissues, liquids, and polymers. It involves placing the end of a coaxial transmission line in contact with the material under test (MUT), creating an impedance mismatch at the probe–sample interface. This mismatch partially reflects the incident electromagnetic wave, whose magnitude and phase (the complex reflection coefficient S_11_) are measured by a VNA over a broadband frequency range. S_11_ is directly influenced by the frequency-dependent permittivity of the MUT.

**Figure 1:**
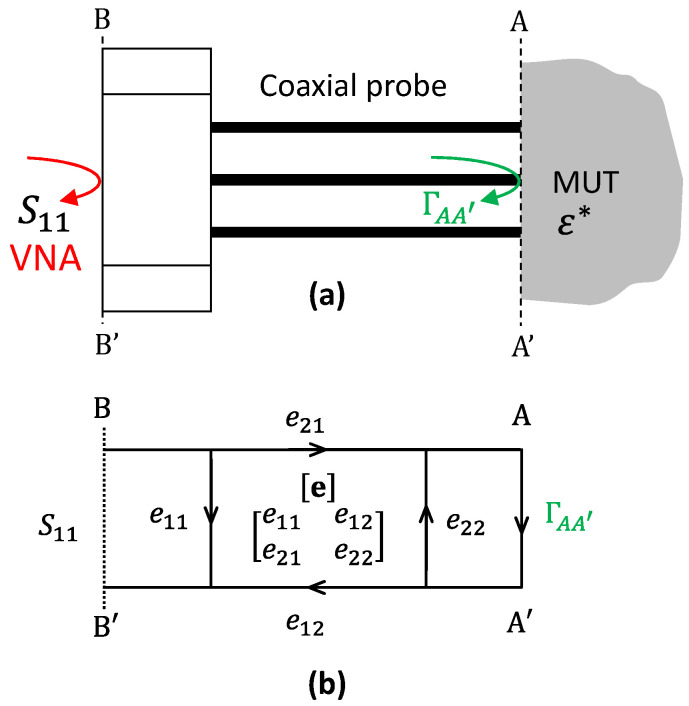
The open-ended coaxial probe. (**a**) A schematic of the measurement system, where the VNA measures the reflection coefficient S_11_, which is affected by the systematic errors introduced by the probe and its interface with the MUT. (**b**) A representation of the coaxial probe’s error-box model, illustrating the relationship between S_11_ and the reflection coefficient at the probe’s aperture, $\Gamma_{AA^{\prime }}$.

As shown in Figure 1a, the raw $S_{11}$ measurement is referenced to the calibration plane BB’ of the VNA, typically located at the connector interface of the probe. The load impedance at this plane, $Y_{BB^{\prime }}$, can be calculated as follows:

(4) $$Y_{BB^{\prime }}=Y_{0}\frac{1-S_{11}}{1+S_{11}},$$

with $Y_{0}=0.02$ Ω^−1^ for a 50-Ω system.

However, to accurately interpret this reflection in terms of the sample’s permittivity, it is necessary to relocate the reference plane to the aperture plane, AA′, where the electromagnetic field actually interacts with the sample. This translation is non-trivial, as it requires accounting for both the systematic measurement errors intrinsic to the VNA and the propagation effects within the probe body. These include directivity errors, an imperfect source/load match, and tracking discrepancies, all of which are modeled using a three-term complex error matrix [e], as in Figure 1b [45].

When a full error model is employed, the reflection coefficient at the probe’s aperture can be recovered through a Möbius transformation [45]:

(5) $$\Gamma_{AA^{\prime }}=\frac{S_{11}-e_{11}}{e_{22}S_{11}-\det\left[e\right]},$$

where $e_{11}$, $e_{22}$, and the determinant of $\left[e\right]$ are complex parameters determined through calibration. The admittance at the aperture is then calculated as follows:

(6) $$Y_{AA^{\prime }}=Y_{0}\frac{1-\Gamma_{AA^{\prime }}}{1+\Gamma_{AA^{\prime }}},$$

This transformation permits interpreting the measured data in terms of physically meaningful admittance values.

To relate this admittance to the $\varepsilon^{*}$ of the MUT, three theoretical models are considered in this work: the capacitive model, the radiation model, and the virtual transmission line model. Each of these models represents a different degree of approximation regarding the electromagnetic behavior near the probe’s aperture and applies distinct assumptions about the probe–sample interaction. In this work, all the models are calibrated using three experimental standards: a short circuit, ambient air, and distilled water. These standards are selected due to their well-characterized or idealized admittance properties, which serve as boundary conditions in model parameterization. The capacitive model supports a full vector error correction with an analytical inversion. By contrast, the radiation and virtual line models, which better represent higher-frequency behavior, use simplified transmission line approximations and numerical inversion. These models consider the probe as a uniform section with a complex propagation constant and characteristic admittance (obtained experimentally with short and air standards), effectively absorbing systematic effects into those line parameters.

#### 2.2.1. Capacitive Model

**Figure 2:**
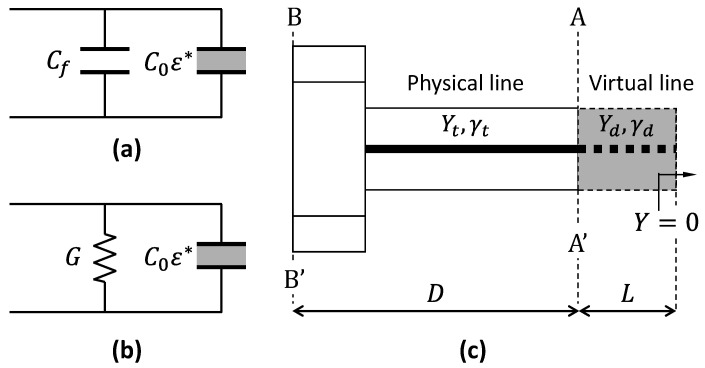
The equivalent circuits for the (**a**) capacitive, (**b**) radiation, and (**c**) virtual line models.

In the capacitive model, it is assumed that there is an evanescent electric field that penetrates into the sample, inducing a capacitive response. As shown in Figure 2a, the load admittance $Y_{AA^{\prime }}$ is given by [46]

(7) $$Y_{AA^{\prime }}=j\omega \left(C_{0}\varepsilon^{*}+C_{f}\right),$$

where $C_{0}$ is a geometry-dependent proportionality constant that describes the coupling between the probe field and the MUT’s permittivity, and $C_{f}$ represents the fringing field in the dielectric of the probe, which is usually much smaller than the $C_{0}$.

To determine $\varepsilon^{*}$, we calibrate the probe with a short circuit, air, and a reference liquid of known permittivity, obtaining admittance and reflection data, $Y_{i}$ and $S_{11}^{i}$, for the $i\in \left\{1,2,3\right\}$. For an unknown (fourth) sample, the admittance, $Y_{4}$, can be derived from its reflection coefficient, $S_{11}^{4}$, using the cross-ratio invariance [5]

(8) $$\frac{\left(Y_{4}-Y_{1}\right)\left(Y_{2}-Y_{3}\right)}{\left(Y_{4}-Y_{2}\right)\left(Y_{3}-Y_{1}\right)}=\frac{\left(S_{11}^{4}-S_{11}^{1}\right)\left(S_{11}^{2}-S_{11}^{3}\right)}{\left(S_{11}^{4}-S_{11}^{2}\right)\left(S_{11}^{3}-S_{11}^{1}\right)}.$$

This relationship allows the determination of the complex admittance of an unknown sample directly from raw $S_{11}$ values, without any explicit knowledge of the error matrix elements.

Because, in this model, there is a linear relationship to $\varepsilon^{*}$, the permittivity of the MUT can be obtained using the following:

(9) $$\varepsilon^{*}=\frac{{∆}_{42}{∆}_{31}}{{∆}_{41}{∆}_{32}}\varepsilon_{ref}^{*}+\frac{{∆}_{43}{∆}_{21}}{{∆}_{41}{∆}_{23}},$$

where ${∆}_{ij}=\left(S_{11}^{i}-S_{11}^{j}\right)$, $i,j\in \left\{1,2,3,4\right\}$. The raw parameters $S_{11}^{1}$, $S_{11}^{2}$, $S_{11}^{3}$, and $S_{11}^{4}$ correspond to the short circuit, air, reference liquid, and MUT, respectively. $\varepsilon_{ref}^{*}$ is the complex permittivity of the reference liquid.

This model typically works best at lower frequencies (or with a small probe diameter), where fringing fields dominate and radiation effects are minimal. Its straightforward and efficient implementation makes it attractive for low-cost systems prioritizing simplicity.

#### 2.2.2. Radiation Model

At higher frequencies, purely capacitive approximations often break down, as the probe’s aperture acts as a radiating structure. The radiation model treats the probe’s aperture as a radiating element, with the MUT represented by an equivalent circuit (e.g., a parallel capacitor and resistor) [47]. In this work, the sample is modeled by a capacitor, $C_{0}\varepsilon^{*}$, plus a frequency-dependent radiation term, $G\left(\omega ,\varepsilon^{*}\right)$. As illustrated in Figure 2b, the total admittance is as follows:

(10) $$Y_{AA^{\prime }}=j\omega C_{0}\varepsilon^{*}+G\left(\omega ,\varepsilon^{*}\right).$$

For an infinitesimal antenna, radiation conductance can be approximated by [47]

(11) $$G\left(\omega ,\varepsilon^{*}\right)={\varepsilon^{*}}^{\frac{5}{2}}G\left(\omega ,\varepsilon_{0}\right),$$

yielding a nonlinear relationship between $Y_{AA^{\prime }}$ and $\varepsilon^{*}$:

(12) $$Y_{AA^{\prime }}=j\omega C_{0}\varepsilon^{*}+{\varepsilon^{*}}^{\frac{5}{2}}G\left(\omega ,\varepsilon_{0}\right).$$

To obtain $Y_{AA^{\prime }}$, we modeled the probe as a transmission line segment of the length, *D*; the characteristic admittance, $Y_{t}$; and the propagation constant, $\gamma_{t}$. The measured admittance, $Y_{BB^{\prime }}$, was then mapped to $Y_{AA^{\prime }}$ by the following:

(13) $$Y_{AA^{\prime }}=Y_{t}\frac{Y_{t}\tanh\gamma_{t}D-Y_{BB^{\prime }}}{Y_{BB^{\prime }}\tanh\gamma_{t}D-Y_{t}},$$

From the short and air standards, we obtained $Y_{BB^{\prime }}^{short}$ and $Y_{BB^{\prime }}^{air}$, via Equation (4). The short standard load admittance is $Y_{AA^{\prime }}^{short}=\infty$, and for air, $Y_{AA^{\prime }}^{air}=j\omega C_{air}$, where $C_{air}=2.38\varepsilon_{0}\left(r_{d}-r_{i}\right)$ [8], with $r_{d}$ and $r_{i}$ being the outer and inner radii, respectively. Using Equation (13) for the short circuit and the air, we solved for $Y_{t}$ and $\tanh\gamma_{t}D$:

(14) $$Y_{t}=\sqrt{Y_{BB^{\prime }}^{air}\cdot Y_{BB^{\prime }}^{short}+j\omega C_{air}\left(Y_{BB^{\prime }}^{air}-Y_{BB^{\prime }}^{short}\right)},$$

(15) $$\tanh\gamma_{t}D=\frac{Y_{t}}{Y_{BB^{\prime }}^{short}},$$

The third standard (reference liquid) was then used to determine $C_{0}$ and G. Once $C_{0}$ and G were known, the permittivity of the MUT was found by numerically solving Equation (10).

A complete error correction analogous to the capacitive model would require a fourth standard, which was not used here because no additional suitable reference was available.

#### 2.2.3. Virtual Line Model

In the virtual line model, the sample is treated as a dielectric-loaded transmission line with an effective length of *L* [48], as shown in Figure 2c. The field in the MUT is assumed to propagate within this virtual coaxial line, which is terminated by an open condition. In practice, no physical extension of the inner or outer conductors is introduced; the virtual line is a mathematical construct that represents the fringing-field region as an equivalent coaxial section of the length, *L*, filled entirely by the MUT. The equivalent admittance is as follows:

(16) $$Y_{AA^{\prime }}=Y_{d}\tanh\gamma_{d}L.$$

where $Y_{d}$ and $\gamma_{d}$ are the characteristic admittance and the complex propagation constant of the virtual line, respectively, defined by the following:

(17) $$Y_{d}\approx \frac{\sqrt{\varepsilon^{*}}}{60\Omega \ln\frac{r_{d}}{r_{i}}}$$

(18) $$\gamma_{d}=j\frac{\omega \sqrt[{\;}]{\varepsilon^{*}}}{c},$$

where *c* is the speed of light in a vacuum. As in the radiation model, $Y_{BB^{\prime }}$ is mapped to $Y_{AA^{\prime }}$, and the reference liquid standard is used to calibrate *L*. Finally, $\varepsilon^{*}$ is determined by numerically inverting Equation (16), which is transcendental in $\varepsilon^{*}$.

## 3. Materials and Methods

This section details the proposed low-cost system, the methodology used for its validation, and a comparison with a widely used commercial probe system to gauge the reliability of the low-cost approach. We characterize reference liquids, saline solutions, and biological tissues, covering a broad spectrum of dielectric properties.

The methodology follows a structured approach, starting with a Short-Open-Load (SOL) calibration to minimize VNA and cabling systematic errors prior to probing. The probe is then connected and placed in contact with each sample, and the reflection coefficient (S_11_) is recorded. We process the data in Python (version 3.10) for the low-cost system, while the commercial system uses proprietary software. Multiple measurements per sample are assessed repeatability, and comparisons with the commercial system and reference values evaluate the accuracy. Factors like probe positioning, air gaps, and ambient conditions are also considered.

### 3.1. Low-Cost System

**Figure 3:**
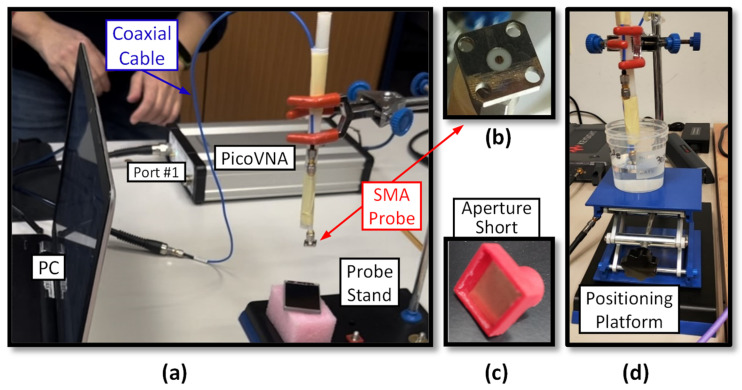
(**a**) The proposed experimental probe system based on an SMA connector and a picoVNA 106. (**b**) A bottom view of the connector used as a probe. (**c**) A short-circuit block for the SMA probe. (**d**) Sample positioning.

The low-cost system employs an SMA coaxial probe connected to a VNA. We use either a PicoVNA 106 (300 kHz–6 GHz; Pico Technology Ltd., St Neots, Cambridgeshire, UK) (in the photo in Figure 3a) or a Keysight Streamline P9374B (9 kHz–20 GHz; Keysight Technologies, Santa Rosa, CA, USA). All the measurements were taken with an intermediate-frequency bandwidth of 3 kHz, a source power of −10 dBm, and 16 trace averages. The SMA probe was built from a panel-mount connector (model R124510000; Radiall SA, Aubervilliers, France), whose center pin was cut and filed flush with its square mounting plane (Figure 3b). The center conductor is ∅_i_ = 1.28 mm in diameter, and the outer conductor’s inner diameter is ∅_d_ = 4.1 mm.

The applicable frequency range of the probe–MUT system is constrained by several factors: (i) the probe’s aperture, which governs the validity of fringing-field approximations versus radiation models [32]; (ii) the dielectric properties of the MUT, particularly the modulus of complex permittivity [17]; (iii) the SNR and the dynamic range of the VNA [9]; and (iv) the insertion loss from cables and connectors, which limits the useful upper frequency. In our system, the SMA probe remains in single-mode operation well beyond 6 GHz. However, as shown in this work, the extraction accuracy can depend strongly on the model used, with no additional benefit observed from virtual line modeling in our frequency range.

The effective sensing volume of the coaxial probe is determined by the spatial extent of the near field at the probe’s tip. The lateral sensing radius—defined as the radial distance from the center, beyond which the electric field becomes negligible—is typically estimated as being 60–70% of the outer conductor’s inner radius [18,32]. For the present SMA probe, this corresponds to approximately 1.3–1.5 mm. To ensure the full containment of the reactive field and minimize edge effects, the sample should extend at least 6 mm × 6 mm laterally. In depth, the sensing range is typically limited to a few millimeters depending on the sample’s permittivity; in our case, a minimum depth of 2 mm was sufficient to avoid interaction with the substrate. These spatial constraints are consistent with those reported in previous studies [49] and are comparable to those of commercial slim-form probes.

To ensure the probe’s stability and prevent movement during sample placement, the cable is taped to a cylindrical plastic support (Figure 3a,d), which can be clamped and vertically adjusted on a metal rod. After each measurement, the probe is cleaned with isopropyl alcohol to avoid cross-contamination.

A custom, Python-based application controls the VNA, automates data acquisition, and performs complex permittivity calculations. Users enter the probe’s geometry (inner/outer radii) and temperature. The software includes a Measurement Mode for real-time acquisition and a Demo Mode for replaying pre-recorded S-parameters (Touchstone files), allowing data from simulations or alternative VNAs/probes to be processed.

During measurement, the sample is raised until it contacts the probe’s open end, ensuring full aperture coverage. Liquids need only submerge the square mounting base (Figure 3d). For elastic solids, it is important to apply enough pressure on the surface to get a good contact [13,50]. If the sample is heterogeneous, multiple spots are measured and averaged.

All the measurements were carried out at 23 °C. For system calibration, a short-circuit block (a copper sheet completely covering the probe opening, as in Figure 3c), air, and distilled water were used as standards, with the water permittivity modeled using the Debye Equation (2) with $\varepsilon_{s}=79.1$, $\varepsilon_{\infty }=5.1$, and τ=8.7 ps [51].

Each measurement session was limited to 1–2 h and was preceded by a full short–air–water calibration. The calibration was repeated if the probe was reconnected, or if more than two hours had elapsed, in order to minimize the impact of ambient temperature fluctuations or cable repositioning. Under these conditions, no appreciable drift was observed in the water standard: the magnitude of S_11_ remained stable to within 1 dB throughout the session. Although a formal study of long-term drift was not performed, the protocol followed ensured consistent calibration stability during typical use.

Excluding the VNA, the complete low-cost probe setup can be assembled for under EUR 100: the SMA connector used as probe body costs ≈EUR 15, the copper plate used as the short standard costs <EUR 1, the RF cables and adapters cost ≈EUR 30, and a basic bench clamp stand costs ≈EUR 30. All the acquisition and data-processing routines were written in Python and added no additional cost

### 3.2. Commercial System

The experimental setup based on a commercial Keysight probe served as a high-precision reference for dielectric characterization. Unlike the low-cost, SMA-based probe, this system provides superior accuracy and stability over an extended frequency range, up to 50 GHz. It is widely used in research for applications requiring highly reliable dielectric measurements, such as biological tissue characterization. As an industry-standard tool, it ensures reproducible and comparable results, allowing for the validation of the low-cost system proposed in this study.

The system consists of a Keysight Slim Probe (N1501A), a high-precision, open-ended coaxial probe designed for dielectric measurements, and a Keysight Streamline VNA: model P9371A (300 kHz–6.5 GHz) and P9374B (9 kHz–20 GHz). The setup includes a positioning platform to ensure precise probe placement, reducing variability in measurements.

The calibration standards used include air, a shorting block designed for this probe, and distilled water. For this measurement system, the manufacturer specifies a guaranteed minimum operating frequency of 500 MHz. The probe’s estimated measurement error is ±10% when used with Keysight’s Materials Measurement Suite [33].

### 3.3. Materials

The materials under test were grouped into three categories: pure polar liquids, saline solutions with variable ionic conductivity, and biological tissues. These groups were chosen to test the system’s response under progressively more complex dielectric scenarios.

The first set comprised methanol, ethanol, acetone, propan-1-ol, and propan-2-ol. These predominantly dipolar liquids have well-documented permittivity spectra [52], making them ideal for verifying both the hardware and algorithms. Comparisons of the results from the SMA probe and the commercial probe against reference data allow us to gauge the measurement accuracy.

The second category consisted of aqueous solutions of sodium chloride (NaCl), prepared at concentrations ranging from 0 to 1.2% *m*/*v*. These represent materials with significant ionic conductivity. Such solutions exhibit complex dielectric responses at lower frequencies due to ionic conduction. They are a standard test sample in dielectric spectroscopy, as dissolved ions and polar water molecules jointly influence permittivity, thus testing our system’s ability to capture both dipolar and conductive contributions. The saline solutions were prepared by heating distilled water to 50 °C, dissolving the salt under stirring until fully dissolved, and allowing the solution to cool to 23 °C.

The third and most complex group comprised biological tissues, including egg white and yolk, chicken breast, and pork liver, loin, skin, and fat. All the samples were acquired from a supermarket, kept refrigerated until the experiment, then brought to room temperature (23 °C) before measurement. Solid tissues were trimmed into portions of approximately 2 cm × 2 cm, with a thickness of at least 1 cm. The egg white and egg yolk were poured into individual polypropylene jars (30 mm inner diameter) to a depth greater than 10 mm. In every case, the contact area exceeded the probe’s aperture by at least a factor of four, and the material depth was sufficient to ensure that the electromagnetic field interacted only with the sample.

These highly heterogeneous, lossy materials have dielectric properties influenced by their water content, ionic makeup, membranes, and macromolecules. Their strong frequency dispersion and spatial variability make accurate characterization challenging, providing a stringent test for biomedical applications.

## 4. Results and Discussion

This section presents the experimental validation of the low-cost measurement system by analyzing the complex permittivity of various materials in the microwave frequency range. The results are compared to those obtained using a commercial reference system in controlled and consistent measurement conditions. We emphasize the evaluation of the accuracy, frequency-dependent behavior, and robustness of the proposed approach when applied to samples with different dielectric properties.

For each material, the measured S_11_ parameters were processed using the three models described in Section 2.2. The corresponding permittivity curves are presented together with commercial system measurements and theoretical data, when available.

To ensure comparability, the measurements were performed using the same acquisition protocol, including repeated measurements and standardized calibration. We discuss the results considering accuracy, model suitability, and reproducibility, highlighting the capabilities and limitations of the low-cost setup in practical scenarios.

An overview of the measurement campaign is summarized in Table 1, which details each sample type, measurement system used, frequency range, and processing method.

### 4.1. Pure Polar Liquids

Experiments were carried out using both the low-cost SMA probe and the commercial system, employing the same VNA (Keysight model P9374B) configured to cover the frequency range from 10 MHz to 15 GHz. The Keysight measurement suite, in addition to providing the permittivity value, also saves the measured S-parameters. Because the commercial software disables measurements on any other port, it was not possible to measure each sample simultaneously with both systems. Consequently, all the measurements were first performed using the commercial probe and subsequently repeated using the low-cost probe, ensuring that the sample temperature remained constant throughout.

For each sample, the reflection coefficient, S_11_, was recorded under carefully controlled conditions, including temperature stability. The S-parameter data from the SMA probe were analyzed using the three theoretical models (the capacitive, radiation, and virtual line models), whereas the data from the commercial system were processed using its dedicated software suite. Reference complex permittivity values from the literature were used for comparative validation.

**Figure 4:**
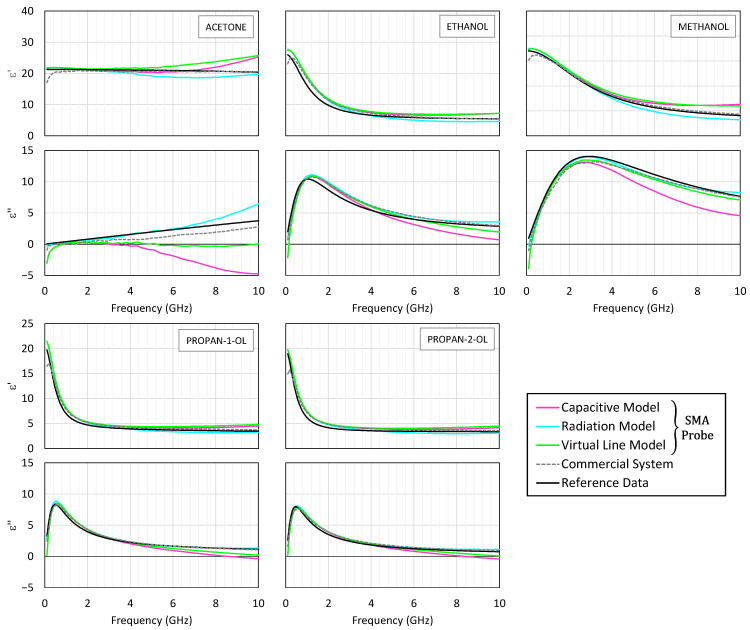
The real part (top panel) and the imaginary part (bottom panel) of the measured permittivity of acetone, ethanol, methanol, propan-1-ol, and propan-2-ol. The measurement results with the SMA probe are shown in continuous pink, blue, and green lines corresponding to the capacitive, radiation, and virtual line models, respectively. The reference data according to [52] is shown in a continuous black line. The results obtained with the commercial probe and its measurement suite are shown in dashed lines.

**Figure 5:**
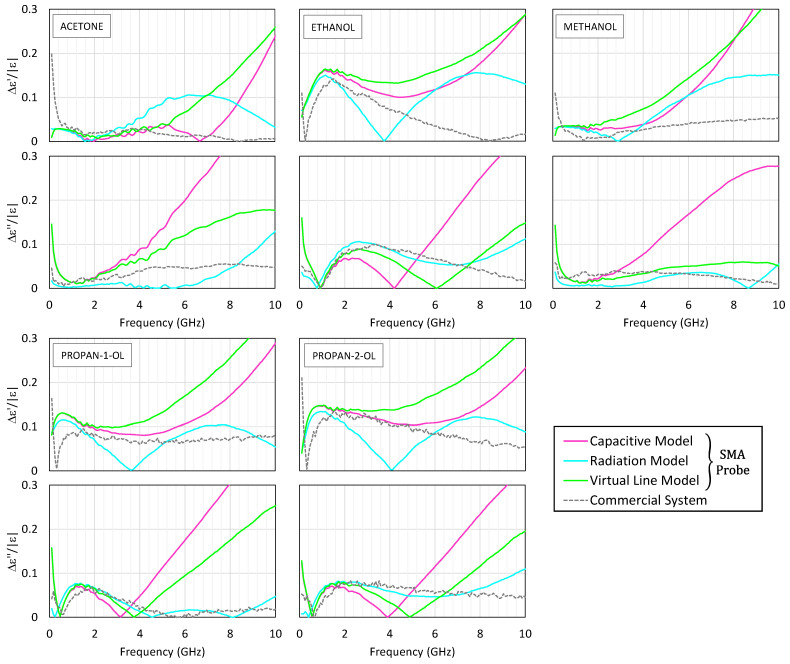
The errors in the real part (top panel) and the imaginary part (bottom panel) of the measured permittivity of acetone, ethanol, methanol, propan-1-ol, and propan-2-ol. The measurement results with the SMA probe are shown in continuous pink, blue, and green lines corresponding to the capacitive, radiation, and virtual line models, respectively. The results obtained with the Keysight probe and its measurement suite are shown in dashed lines.

Figure 4 presents the permittivity spectra obtained for the five tested liquids. The real and imaginary parts are plotted as functions of frequency, with the results from the three SMA-based models shown in solid lines, the commercial system results in dashed lines, and the reference data in black. For clarity and to maintain consistency with the valid operating range of the low-cost system, only the results up to 10 GHz are shown in both Figure 4 and Figure 5, since beyond this frequency, the SMA probe exhibits significant deviations and no longer provides reliable data.

Figure 5 shows the corresponding error plots, depicting the relative deviations of both the real and the imaginary parts, with respect to the reference values. The relative errors shown in Figure 5 are computed as follows:

(19) $${∆}_{\varepsilon^{\prime }}={\left|\frac{\varepsilon_{m}^{\prime }\left(f\right)-\varepsilon_{t}^{\prime }\left(f\right)}{\left|\varepsilon_{t}^{*}\left(f\right)\right|}\right|}_{max},$$

(20) $${∆}_{\varepsilon^{\prime \prime }}={\left|\frac{\varepsilon_{m}^{\prime \prime }\left(f\right)-\varepsilon_{t}^{\prime \prime }\left(f\right)}{\left|\varepsilon_{t}^{*}\left(f\right)\right|}\right|}_{max}.$$

The subscripts “*m*” and “*t*” denote the measured and the reference values, respectively.

Up to about 2.5 GHz, the three models for the SMA probe exhibit close agreement, with the capacitive and virtual line models producing almost indistinguishable results. However, at higher frequencies, the capacitive model begins to deviate, particularly in estimating dielectric losses, sometimes resulting in negative values for the imaginary part—an indication of model breakdown. In contrast, the radiation model remains stable and accurate up to 10 GHz, closely matching the commercial system’s output and with a relative error typically below 10%.

To complement the graphical comparison, Table 2 reports the relative errors of the extracted *ε*′ and *ε*″ values for the polar liquids against the literature-reported values at 0.5, 5, and 10 GHz. These values are computed using Equations (19) and (20).

Acetone, being intrinsically low loss, demonstrates how small perturbations can significantly affect the imaginary permittivity component. Both systems show noticeable deviations in this case. Additionally, below 500 MHz, the commercial system shows inconsistencies, aligned with the manufacturer’s stated operational floor.

**Figure 6:**
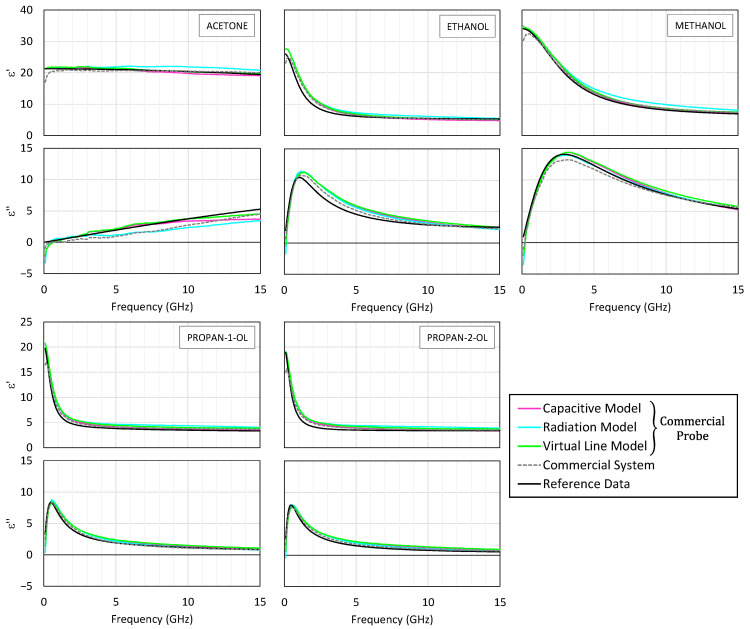
The results obtained with the commercial probe. The real part (top panel) and the imaginary part (bottom panel) of the measured permittivity of acetone, ethanol, methanol, propan-1-ol, and propan-2-ol. The results processed with the capacitive, radiation, and virtual line models are shown in continuous pink, blue, and green lines, respectively. The theoretical value according to [52] is shown in a continuous black line. The results obtained with the commercial probe and its measurement suite are shown in dashed lines.

To decouple model behavior from probe geometry, the S_11_ measurements from the commercial probe were reprocessed using the demo mode of the custom Python application. In this analysis, the probe was modeled as a coaxial structure equivalent to an RG-405 line (inner conductor diameter ∅_i_ = 0.57 mm; outer conductor inner diameter ∅_d_ = 1.68 mm) [15]. As shown in Figure 6, the capacitive model most closely aligns with the commercial software across the tested frequencies, while the radiation model displays larger deviations, especially at lower frequencies. Interestingly, for frequencies below 500 MHz, the custom application often produced values closer to theoretical references than the proprietary suite. Table 3 summarizes the relative deviation of *ε*′ and *ε*″ between the values obtained by reprocessing the commercial probe data with the three analytical models and those returned by the vendor’s proprietary software, evaluated at 0.5, 5, 10, and 15 GHz.

These findings suggest that the capacitive model is particularly suitable when the probe geometry is similar to that of the commercial device and maintains an acceptable accuracy up to 15 GHz in such cases. For the low-cost SMA probe, however, the radiation model appears more robust at higher frequencies, despite using only three standards for calibration. The virtual line model, while reasonable, does not offer a clear advantage over the other two. Consequently, model selection should consider not only the MUT’s expected permittivity range and the frequency band but also the probe’s geometry. Matching model and probe characteristics can enhance the reliability of permittivity retrieval, especially under low-cost hardware constraints.

### 4.2. Saline Solutions

The measurements were conducted with both the commercial and the low-cost SMA probes under identical conditions, using the Keysight P9374B VNA. Only the capacitive model was applied here, given that the test samples (distilled water with varying NaCl concentrations) closely resemble the high-frequency behavior of the reference liquid, while exhibiting dominant ionic conduction at lower frequencies.

**Figure 7:**
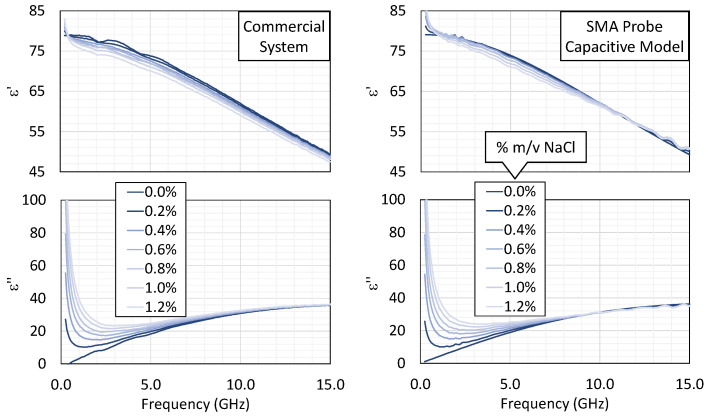
The variations in $\varepsilon^{\prime }$ (**top**) and $\varepsilon^{\prime \prime }$ (**bottom**) as a function of frequency for different NaCl concentrations (% *m*/*v*). A comparison between the results of the commercial system and those of the custom system (capacitive model only).

Figure 7 displays the permittivity spectra obtained for NaCl concentrations ranging from 0 to 1.2% *m*/*v*. Increasing the NaCl content leads to a gradual decrease in the real part of the permittivity, $\varepsilon^{\prime }$, due to hydration shells restricting water molecule rotation [54,55]. At the same time, the imaginary component, $\varepsilon^{\prime \prime }$, increases as a result of the enhanced ionic conductivity. This behavior is well described by the extended Cole–Cole model with an added conductive term [44].

With the commercial probe, the permittivity computed by the capacitive model closely matches the proprietary suite’s results, confirming the correctness of the numerical approach. The SMA probe likewise captures the main trends well, although above ~10 GHz, the curves for different concentrations converge somewhat, indicating the limited sensitivity of the low-cost hardware in resolving small differences in permittivity.

**Figure 8:**
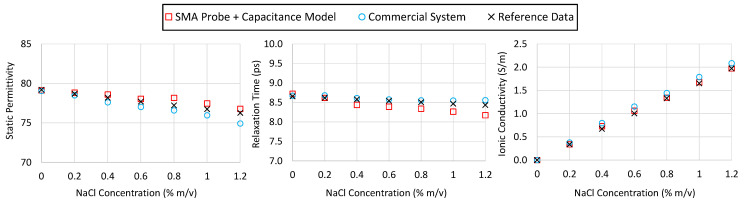
The variation in the dielectric parameters with the NaCl concentration: static permittivity, relaxation time, and ionic conductivity as a function of NaCl concentration. The values were obtained by fitting the Cole–Cole equation to measurements from the commercial system (circles) and the custom SMA-based probe using the capacitance model (squares). Reference values (cross markers) from [44] are also shown.

To confirm these observations, the experimental spectra were fitted to the extended Cole–Cole equation, yielding the static permittivity, $\varepsilon_{s}$, relaxation time, τ, and ionic conductivity, $\sigma_{i}$. The high-frequency permittivity, $\varepsilon_{\infty }$, was fixed to the value for pure water at 23 °C, and the dispersion broadening parameter, α, was set to zero. This simplification assumes a single dominant relaxation process and avoids overfitting in the presence of limited spectral resolution or noise. Figure 8 compares the extracted dielectric parameters as a function of the NaCl concentration. The results obtained from the measurements with the custom SMA-based probe and the commercial system are shown, along with reference values from [44], which are used as reference values in the literature. All three sources exhibit the same trends: As expected, $\varepsilon_{s}$ and τ decrease with increasing salinity, reflecting reduced dipolar mobility. In contrast, $\sigma_{i}$ exhibits a nearly linear increase due to the greater availability of charge carriers.

Overall, the results show that the low-cost system, despite having simpler hardware, effectively captures the salient dielectric features in saline solutions, with strong agreement with the commercial reference up to about 10 GHz.

### 4.3. Biological Tissues

The measurements were carried out using both the commercial probe and the low-cost SMA probe, but with different VNAs to ensure that measurements with both systems could be performed on each sample within the shortest possible time window, thereby minimizing the degradation of biological tissues due to environmental exposure. The commercial probe was connected to a Keysight P9371A (10 MHz–6.5 GHz), while the SMA probe was paired with the PicoVNA 106 (10 MHz–6 GHz). This dual-VNA configuration also enabled the secondary goal of validating the permittivity measurements using a more affordable VNA alternative to the Keysight system.

Each tissue was measured at three points to account for spatial heterogeneity and to obtain statistically representative permittivity values. The results were compared against both the IT’IS Foundation’s parametric models for the corresponding human tissues [53], when available, and the measurements obtained with the commercial system using its proprietary analysis suite.

**Figure 9:**
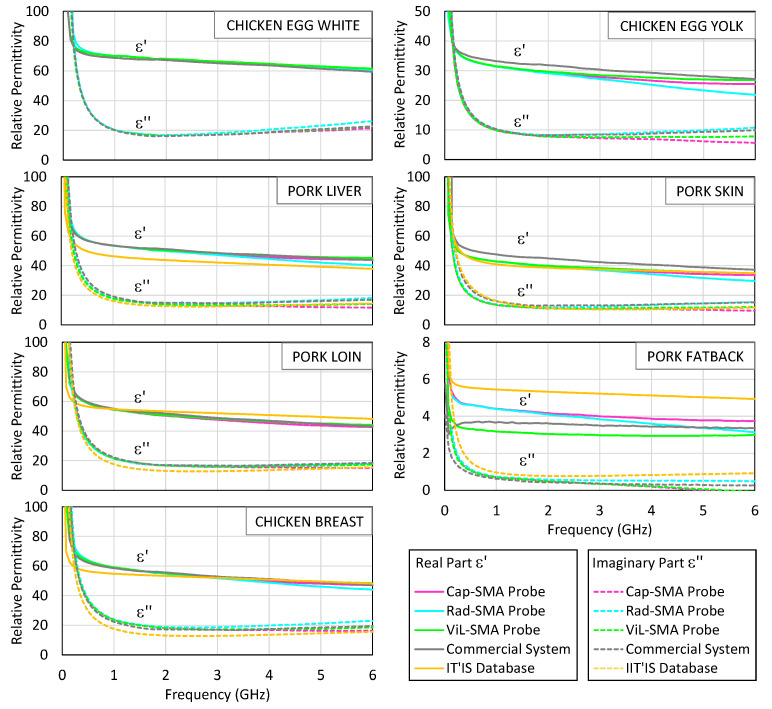
The real part (solid line) and the imaginary part (dashed line) of the measured permittivity of egg white, egg yolk, pork liver, pork skin, pork loin, pork fat, and chicken breast. The measurement results with the SMA probe are shown in pink, blue, and green lines corresponding to the capacitive, radiation, and virtual line models, respectively. The results obtained with the Keysight probe and its measurement suite are shown in grey lines. The values recorded in the IT’IS Foundation database for homologous human tissues [53] are shown in orange lines.

Figure 9 displays the complex permittivity spectra (real and imaginary parts) for various biological tissues measured with the SMA-based probe using the three theoretical models, along with results from the commercial probe and IT’IS reference data. The tissues rich in water and electrolytes (e.g., egg white, muscle) showed high values of both $\varepsilon^{\prime }$ and $\varepsilon^{\prime \prime }$, whereas the lipid-dense tissues (e.g., fat) displayed markedly lower values, reflecting reduced dipolar relaxation and lower ionic conductivity. The imaginary component $\varepsilon^{\prime \prime }$ is reasonably well captured by the radiation model in most of the samples, especially above 2–3 GHz, where dielectric losses dominate. However, for the real part, $\varepsilon^{\prime }$, the performance is more variable: although the radiation model provides acceptable results, it does not systematically yield better estimates than the capacitive or virtual line models. In some tissues, such as egg white or liver, these alternative models yield values that more closely match the commercial reference at lower frequencies.

Table 4 reports the maximum relative errors of *ε*′ and *ε*″ across the 0.5–6 GHz band for each tissue sample extracted using the SMA-based system. These values provide a compact quantitative summary of the model’s performance beyond the visual interpretation of Figure 9. It is noted that while the real part of the low-loss dielectrics can generally be estimated, the accuracy of *ε*″ is limited by the system noise and model sensitivity; in contrast, high-permittivity tissues are well characterized, as demonstrated by the agreement with the commercial system’s results.

**Figure 10:**
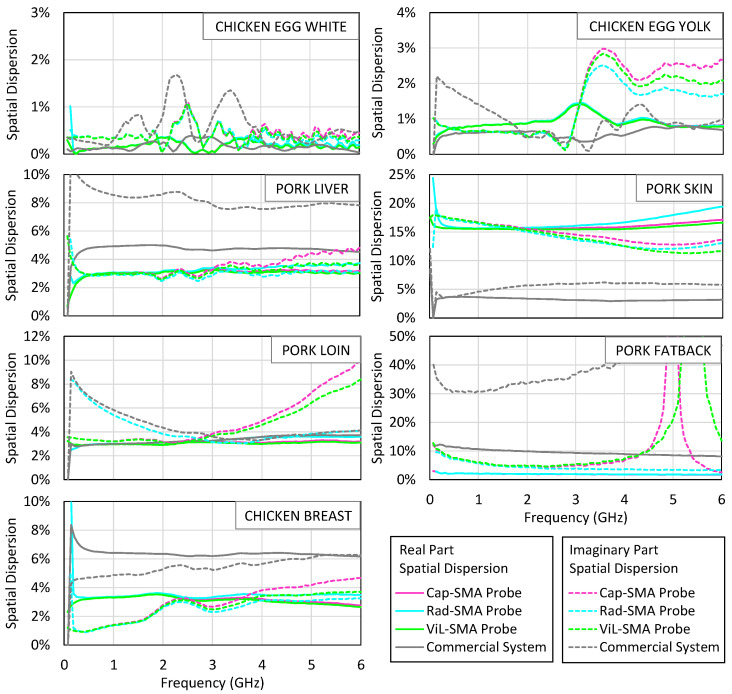
The spatial dispersion of the real part (solid line) and the imaginary part (dashed line) of the measured permittivity of egg white, egg yolk, pork liver, pork skin, pork loin, pork fat, and chicken breast. The measurement results with the SMA probe are shown in pink, blue, and green lines corresponding to the capacitive, radiation, and virtual line models, respectively. The results obtained with the commercial system are shown in grey lines. The dispersion was computed as the maximum deviation from the mean across three measurements, normalized by the mean.

Figure 10 examines the spatial dispersion observed in the three repeated measurements performed for each tissue and each probe. The plotted value corresponds to the maximum difference between any individual measurement and the average, which is then normalized by that average, and computed over the 0.01–6 GHz frequency range. This metric reflects how sensitive each system is to probe repositioning and local surface variability. Most of the tissues show a spatial variation below 10% with both systems, indicating satisfactory mechanical repeatability. The most notable exception is pork skin, where the SMA-based system exhibits a dispersion exceeding 15%, likely due to reduced softness and surface irregularities that hinder uniform contact. The average value measured for pig skin with the SMA probe was also significantly lower than that obtained using the commercial probe, possibly due to air gaps between the probe’s aperture and the skin surface.

In contrast, smoother or more compliant tissues, such as yolk or muscle, yield variations below 5%, indicating that reliable repeatability is achievable when coupling conditions are favorable.

Despite these challenges, the SMA probe enabled the identification of dielectric differences between the tissue types. The accuracy of the permittivity measurements depends critically on the quality of the contact between the coaxial probe and the sample. Air gaps or irregular interfaces can distort the reflection coefficient and introduce systematic errors in the extracted permittivity. This issue is particularly relevant when measuring rough or inhomogeneous surfaces. Applying slight pressure or averaging over multiple locations can help mitigate these effects.

**Figure 11:**
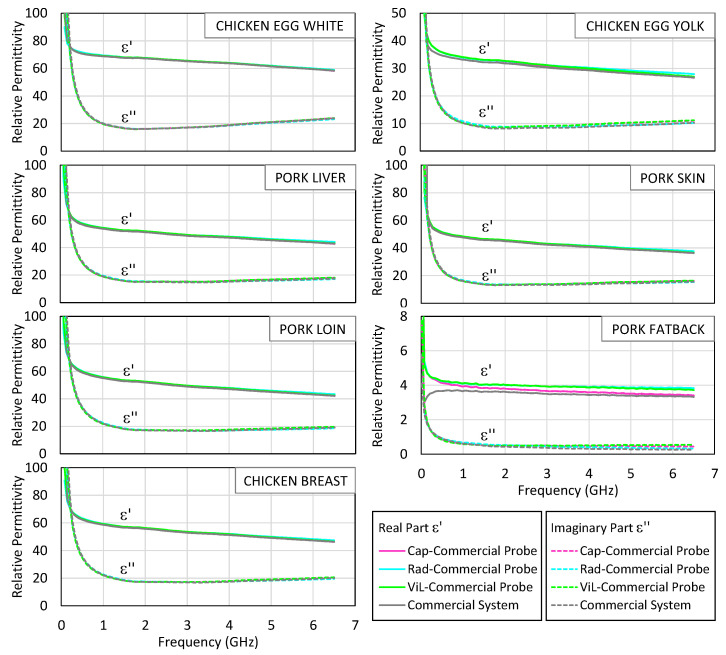
The results obtained with the commercial probe. The real part (solid line) and the imaginary part (dashed line) of the measured permittivity of egg white, egg yolk, pork liver, pork skin, pork loin, pork fat, and chicken breast. The results processed with the capacitive, radiation, and virtual line models are shown in pink, blue, and green lines, respectively. The results obtained with the Keysight probe and its measurement suite are shown in grey.

To further assess model behavior, Figure 11 explores how the three theoretical models perform when applied to the S_11_ measurements obtained with the commercial probe. The resulting permittivity spectra are compared to those generated by the proprietary software of the same probe. This evaluation aims to assess the model’s performance independently of hardware limitations. For *ε*″, the radiation model generally yields values closer to the commercial reference across most tissues and frequencies, particularly in samples with higher dielectric losses. For *ε*′, however, the agreement varies depending on the tissue and frequency range: in some cases, the capacitive or virtual line models provide slightly better alignment. These results suggest that while no single model is universally optimal, the radiation model tends to provide robust estimates of *ε*″, and that model selection should take into account the material properties and measurement priorities.

Apart from the case of pork fat, the curves generated by the custom models are in very close agreement with the commercial software up to 6.5 GHz, confirming the correct implementation of the algorithms. In the case of pork fat, larger discrepancies appear due to the low permittivity of this tissue, which makes accurate estimation more challenging for open-ended coaxial probes. It can be seen that the radiation model provides the closest match to the commercial measurements, especially above 2 GHz. The capacitive model underestimates the imaginary part in this range, while the virtual line model exhibits moderate deviation in both components. This example reinforces the notion that the performance of each model is frequency-dependent and material-specific, and it highlights the superiority of the radiation model in capturing the complex dielectric behavior of soft tissues within the bandwidth of the low-cost system.

These observations reinforce the suitability of the radiation model for biological tissues when using the low-cost probe, particularly in contexts where the conductivity is moderate and dielectric losses are non-negligible. The results also highlight the need for careful model selection based on both the material properties and the operating frequency, especially when working with simplified or cost-constrained measurement setups.

The experimental findings demonstrate the potential of the low-cost configuration for the dielectric characterization of biological tissues, particularly in applications that prioritize material differentiation over absolute accuracy. The results also highlight the importance of selecting the appropriate theoretical model depending on tissue composition, frequency range, and probe quality.

### 4.4. Reproducibility

Reproducibility measurements were performed using three SMA probes manufactured in the same way. To evaluate the reproducibility of the proposed low-cost system, a set of experiments was conducted using the same liquid samples and acquisition conditions. The SMA probes were labeled #1, #2, and #3 and were connected sequentially to the VNA to measure the same set of samples: ethanol, methanol, propan-2-ol, and acetone.

In its final configuration, the low-cost system is intended to operate with a budget-friendly VNA. For this reason, the PicoVNA 106 was selected for these reproducibility tests, conducted over the 0.01–6 GHz range under identical environmental and procedural conditions. A standard SOL calibration was performed prior to connecting each probe. This ensured that the comparison among probes was not biased by a calibration drift or operator variability. The reproducibility was evaluated by computing, for each frequency point, the maximum deviation of the real and imaginary parts of the complex permittivity obtained from the three different SMA probes, relative to the mean value of the three measurements. These values were then normalized by the magnitude of the complex mean permittivity, $\left|\varepsilon^{*}\right|$:

(21) $${∆}_{\varepsilon^{\prime }}=\frac{\underset{1\leq i\leq 3}{\max}\left|\varepsilon_{i}^{\prime }-\langle \varepsilon^{\prime }\rangle \right|}{\left|\varepsilon^{*}\right|}\;\;,\;\;\;\;\;\;\;\;\;\;\;\;\;\;{∆}_{\varepsilon^{\prime \prime }}=\frac{\underset{1\leq i\leq 3}{\max}\left|\varepsilon_{i}^{\prime \prime }-\langle \varepsilon^{\prime \prime }\rangle \right|}{\left|\varepsilon^{*}\right|},\;\;$$

where $\langle \varepsilon^{\prime }\rangle =\frac{1}{3}\left(\varepsilon_{1}^{\prime }+\varepsilon_{2}^{\prime }+\varepsilon_{3}^{\prime }\right)\;$, $\langle \varepsilon^{\prime \prime }\rangle =\frac{1}{3}\left(\varepsilon_{1}^{\prime \prime }+\varepsilon_{2}^{\prime \prime }+\varepsilon_{3}^{\prime \prime }\right),$ and $\left|\varepsilon^{*}\right|=\sqrt{{\langle \varepsilon^{\prime }\rangle }^{2}+{\langle \varepsilon^{\prime \prime }\rangle }^{2}}$.

**Figure 12:**
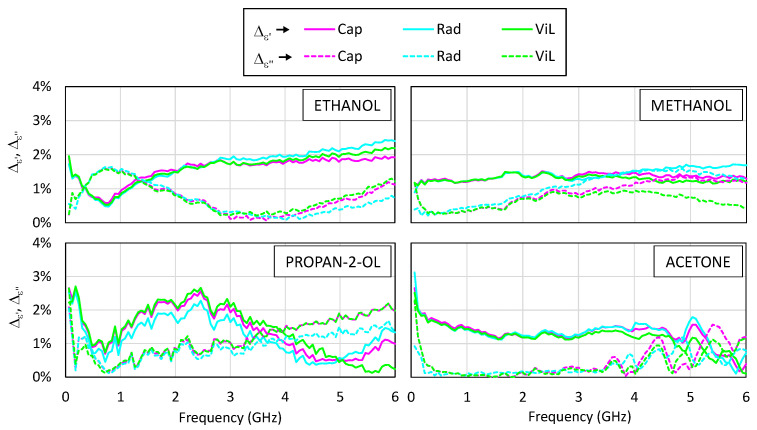
The reproducibility of the complex permittivity measurements for different reference liquids (ethanol, methanol, propan-2-ol, and acetone) obtained with three low-cost SMA probes. The graph shows the maximum normalized deviation from the average value, represented as a function of frequency for the three models used: capacitive (Cap), radiation (Rad), and virtual line (ViL). The continuous lines correspond to the real part, ${∆}_{\varepsilon^{\prime }}$, and the dashed lines correspond to the imaginary part, ${∆}_{\varepsilon^{\prime \prime }}$.

Figure 12 displays the reproducibility results for the four tested polar liquids. It can be observed that, in general, the deviations remain below 3% over the entire frequency range, with most of the values falling under 2% across most of the frequency range. The highest deviations were observed at the extremes of the frequency band, particularly below 0.3 GHz and above 5 GHz, where small changes in the probe’s geometry or mounting may have had a larger impact on the measured response.

Moreover, the trends were preserved regardless of the processing model used. Although minor differences were observed in the magnitude of the deviations depending on whether the capacitive, radiation, or virtual line model was applied, these differences did not exceed 1% between the models in most cases. This suggests that the reproducibility of the hardware is not significantly influenced by the model choice, reinforcing the robustness of the measurement chain as a whole.

These results confirm that the mechanical construction method is sufficiently robust and that the electromagnetic behavior is reproducible across different units.

To further assess the robustness of the system, a reproducibility study was conducted using four polar liquids (acetone, ethanol, methanol, and propan-2-ol). Each liquid was measured a total of seven times: five measurements were taken over a four-month interval using the same probe (SMA #1), with each session preceded by a fresh short–air–water calibration and performed, when available, on a different VNA (PicoVNA 106, Keysight P9374B, or Agilent PNA E8363B). Two additional measurements—one with probe SMA #2 and one with SMA #3—were included to evaluate probe-to-probe and VNA-to-VNA reproducibility under identical conditions. Table 5 summarizes the relative standard deviation (RSD) of the real and imaginary permittivity components, normalized to the complex magnitude $\left|\varepsilon^{*}\right|$. All the values remained below 3% (*ε*′) and 4% (*ε*″) across the 0.05–6 GHz band and all the analytical models, supporting the overall repeatability and stability of the proposed system under typical laboratory conditions. Because the largest RSD is only 3.6%, any change in $\left|\varepsilon^{*}\right|$ greater than roughly 4% rises above this statistical uncertainty and can, therefore, be detected with confidence by the low-cost setup.

## 5. Conclusions

This work presents the validation of a low-cost dielectric characterization system based on an SMA coaxial connector and a compact vector network analyzer, supported by a custom Python-based tool implementing three electromagnetic models: capacitive, radiation, and virtual line. The proposed setup was evaluated using samples of increasing complexity—pure polar liquids, saline solutions, and biological tissues—across the frequency range of at least 0.01–6 GHz.

A clear distinction was observed in the performance of the electromagnetic models depending on probe geometry. For the commercial probe, which features a narrower aperture and reduced fringing fields, the capacitive model proved sufficiently accurate across a test range of 0.01–15 GHz. Its analytical simplicity, invertibility, and stability make it robust and efficient, particularly for clinical or industrial settings requiring quick and consistent measurements. In contrast, for the custom SMA-based probe—characterized by a wider geometry and greater radiation losses—the capacitive model showed increasing discrepancies above ~3–4 GHz, especially with lossy or conductive materials. In this case, the radiation model offered superior accuracy, particularly in reproducing the imaginary part of the permittivity (*ε*″) in conductive samples. The virtual line model provided a stable, but generally intermediate, performance.

To evaluate hardware dependencies, the SMA probe was tested with both a high-end Keysight P9374B VNA and a low-cost PicoVNA 106 for biological tissue samples. The results obtained using the latter, combined with the radiation model, showed a high degree of agreement with the commercial system, validating the proposed low-cost configuration for practical broadband dielectric spectroscopy.

The reproducibility of the low-cost setup was assessed through measurements performed with three independently assembled SMA probes. The variation in extracted permittivity remained below 3% across most of the frequency range, demonstrating mechanical robustness and repeatability in fabrication, calibration, and data processing up to 6 GHz.

While some commercial systems offer broader frequency spans, it is worth noting that the 0.01–6 GHz range covered by the low-cost setup is sufficient for the dielectric characterization of biological tissues, particularly in the context of microwave medical imaging applications, such as early-stage breast tumor detection [56].

These findings can guide model selection depending on the use case. The capacitive model is recommended for low-to-moderate frequencies (up to ≈3 GHz) and moderate permittivity values, especially with narrow-aperture probes. The radiation model provides better accuracy in lossy media or at higher frequencies, as it accounts for energy loss due to radiation. Although the virtual line model is formally more general, it requires solving a transcendental equation for each frequency point, which makes it computationally more intensive than the other two. In our implementation, this model did not provide better accuracy relative to the radiation model for the frequency range and materials tested, neither for the SMA probe nor the commercial probe. This suggests that, in practice, the added complexity does not necessarily translate into improved performance for typical dielectric characterization tasks. Its use may be warranted in scenarios requiring theoretical completeness or a transmission line analysis.

In summary, this study demonstrates that a carefully calibrated, model-informed, low-cost coaxial probe system can provide reliable broadband permittivity measurements. The architecture is well suited for environments requiring affordability, portability, and adaptability—such as teaching labs, field diagnostics, or low-resource research settings. Future developments may focus on extending the frequency coverage, automating calibration, or tailoring the setup to specific clinical or industrial applications. This represents a hardware cost reduction of more than 95% compared to typical commercial solutions, which exceed EUR 2000 for the probe alone and approximately EUR 10,000 including proprietary software and accessories.

## Figures and Tables

**Table 1:** A summary of the samples, probes, frequency range, and processing methods, as well as comparisons with the existing literature.

Material Type	Samples	Probe (VNA)	Frequency Range (GHz)	Data Processing	Comparison with the Literature (Reference)
Pure polar liquids	Methanol Ethanol Acetone Propan-1-ol Propan-2-ol	Commercial probe (P9374B)	0.01–15	Commercial Suite Custom App (3 models)	Yes ([52])
		SMA#1 probe (P9374B)	0.01–15	Custom App (3 models)	Yes ([52])
		SMA#1, #2, #3 probes (picoVNA, P9374B, PNA E8363B) (*)	0.01–6	Custom App (3 models)	Not required
Saline solutions	0–1.2% *m*/*v* NaCl	Commercial probe (P9374B)	0.01–15	Commercial Suite Custom App (**)	Yes ([44])
		SMA#1 probe (P9374B)	0.01–15	Custom App (**)	Yes ([44])
Biological tissues	Egg white, yolk, pork liver, muscle, skin, fat, chicken breast	Commercial probe (P9371A)	0.01–6.5	Commercial Suite Custom App (3 models)	Yes ([53])
		SMA#1 probe (picoVNA)	0.01–6	Custom App (3 models)	Yes ([53])

(*) The Agilent PNA E8363B, as well as probes SMA #2 and SMA #3, were used exclusively to evaluate reproducibility. (**) Capacitive model only.

**Table 2:** The relative error (%) in the real (*ε*′) and imaginary (*ε*″) components of the permittivity for polar liquids, extracted using the SMA-based system. Reference values from the literature [52] were used as the ground truth. Errors were reported at 0.5, 5, and 10 GHz and calculated using Equations (19) and (20).

Liquid	Model	${∆}_{\varepsilon^{\prime }}/{∆}_{\varepsilon^{\prime \prime }}$ 0.5 GHz	${∆}_{\varepsilon^{\prime }}/{∆}_{\varepsilon^{\prime \prime }}$ 5 GHz	${∆}_{\varepsilon^{\prime }}/{∆}_{\varepsilon^{\prime \prime }}$ 10 GHz
Acetone	Capacitive Radiation Virtual Line	2.9/3.1 2.8/0.5 2.9/3.2	3/13.3 8.3/0.2 3.9/8.9	23.7/41.0 3.2/12.9 25.8/17.7
Ethanol	Capacitive Radiation Virtual Line	11.3/5.2 10.6/2.2 11.3/5.2	10.2/5.0 7.2/6.8 13.9/3.5	28.9/35.3 13.0/11.3 28.8/14.8
Methanol	Capacitive Radiation Virtual Line	3.4/3.2 3.3/0.9 3.5/3.2	6.0/12.2 7.3/2.8 10.1/4.6	40.2/27.7 15.0/5.4 34.6/5.1
Propan-1-ol	Capacitive Radiation Virtual Line	13.0/0.4 11.3/3.1 12.9/0.2	8.7/10.9 5.7/0.7 13.1/5.3	28.8/41.3 5.4/4.7 37.5/25.3
Propan-2-ol	Capacitive Radiation Virtual Line	13.1/1.7 11.4/1.0 13.1/1.8	10.4/5.6 4.6/4.8 14.8/0.2	23.3/33.8 8.8/11.0 32.6/19.5

**Table 3:** The relative error (%) in the real (*ε*′) and imaginary (*ε*″) components of the permittivity obtained by reprocessing the commercial probe measurements using the three analytical models. The values returned by the vendor’s proprietary suite were used as references. The errors were evaluated at 0.5, 5, 10, and 15 GHz using Equations (19) and (20).

Liquid	Model	${∆}_{\varepsilon^{\prime }}/{∆}_{\varepsilon^{\prime \prime }}$ 0.5 GHz	${∆}_{\varepsilon^{\prime }}/{∆}_{\varepsilon^{\prime \prime }}$ 5 GHz	${∆}_{\varepsilon^{\prime }}/{∆}_{\varepsilon^{\prime \prime }}$ 10 GHz	${∆}_{\varepsilon^{\prime }}/{∆}_{\varepsilon^{\prime \prime }}$ 15 GHz
Acetone	Capacitive Radiation Virtual Line	6.3/0.7 6.8/1.2 6.8/0.8	1.9/5.4 5.8/1.1 3.5/5.9	3.5/3.2 6.7/1.8 0.8/5.0	4.6/4.0 3.4/5.2 1.2/0.0
Ethanol	Capacitive Radiation Virtual Line	5.5/0.2 5.5/1.5 5.9/0.3	0.1/7.6 8.9/5.2 3.7/8.8	3.8/4.9 12.6/3.0 2.0/8.9	4.4/1.7 7.9/2.0 2.5/4.3
Methanol	Capacitive Radiation Virtual Line	4.3/0.2 4.4/1.3 4.5/0.3	0.2/5.9 6.5/3.1 2.0/6.5	3.8/2.9 10.1/2.4 0.5/5.9	3.2/3.8 7.1/2.8 0.9/1.7
Propan-1-ol	Capacitive Radiation Virtual Line	7.9/0.9 7.6/2.6 8.7/0.7	1.3/7.9 12.6/5.0 8.1/9.8	2.7/6.1 15.6/2.6 6.0/10.9	4.6/0.4 9.5/1.1 5.2/6.5
Propan-2-ol	Capacitive Radiation Virtual Line	8.3/1.1 8.1/2.8 9.2/0.9	1.4/7.9 13.1/5.0 8.7/9.9	2.5/6.2 16.1/2.5 6.7/11.1	4.5/0.7 9.8/0.9 5.7/6.8

**Table 4:** The maximum relative error (%) in the real (*ε*′) and the imaginary (*ε*″) components of the permittivity for each biological tissue sample. The results were computed over the frequency band from 0.5 to 6 GHz for the three analytical models using Equations (19) and (20). The values returned by the vendor’s proprietary suite were used as references.

Tissue	${∆}_{\varepsilon^{\prime }}$ (max)	${∆}_{\varepsilon^{\prime \prime }}$ (max)	${∆}_{\varepsilon^{\prime }}$ (max)	${∆}_{\varepsilon^{\prime \prime }}$ (max)	${∆}_{\varepsilon^{\prime }}$ (max)	${∆}_{\varepsilon^{\prime \prime }}$ (max)
	Capacitive model		Radiation model		Virtual Line Model	
Egg white	2.2%	2.3%	2.8%	5.4%	3.2%	1.2%
Egg yolk	8.8%	15.0%	18.5%	2.9%	6.9%	7.3%
Pork liver	2.8%	11.4%	7.4%	3.9%	3.0%	6.2%
Pork skin	11.6%	14.0%	10.9%	8.3%	18.9%	7.1%
Pork loin	4.1%	8.8%	1.6%	2.4%	3.5%	3.9%
Pork fatback	25.5%	13.9%	25.1%	7.2%	15.7%	10.3%
Chicken breast	2.8%	6.3%	6.4%	7.1%	2.2%	2.8%

**Table 5:** The mean and maximum relative standard deviation (RSD, %) in the real (*ε*′) and the imaginary (*ε*″) parts of the permittivity, computed over the 0.05–6 GHz range for four polar liquids. Each dataset includes seven measurements: five taken with probe SMA #1 over a four-month period and two additional ones taken with SMA #2 and SMA #3. Values are provided for each of the three analytical models.

Liquid	Model	${RSD}_{\varepsilon^{\prime }\%}$ (Mean/Max)	${RSD}_{\varepsilon^{\prime \prime }\%}$ (Mean/Max)
Acetone	Capacitive Radiation Virtual Line	0.9/1.4 1.0/1.5 0.9/1.4	0.6/2.7 0.6/1.4 0.5/2.8
Ethanol	Capacitive Radiation Virtual Line	2.1/2.4 2.4/2.7 2.2/2.4	2.0/2.8 1.6/2.1 1.9/2.5
Methanol	Capacitive Radiation Virtual Line	1.1/1.6 1.3/2.6 1.1/1.6	1.0/2.8 0.9/1.4 0.9/2.8
Propan-2-ol	Capacitive Radiation Virtual Line	2.8/3.6 2.7/3.6 2.9/3.8	1.7/3.2 1.6/2.0 1.6/3.6

## Data Availability

Data are contained within the article.
